# Physicochemical, Functional, Sensory Properties and Willingness to Consume Porridge Made From Composite Flours of Maize, Soybean, and Ripe Plantain

**DOI:** 10.1002/fsn3.71585

**Published:** 2026-03-02

**Authors:** Gifty Serwaa Otoo, Nazir Kizzie‐Hayford, Rosemond Godbless Dadzie, Salifu Seidu‐Larry, Claudia Asantewaa Gyimah, Vivianne Geraldo, Sandra Ama Kaburi, Francis Padi Lamptey, Isaaca Adade, Jerry Ampofo‐Asiama

**Affiliations:** ^1^ Department of Food Science and Postharvest Technology Cape Coast Technical University Cape Coast Ghana; ^2^ Department of Biochemistry University of Cape Coast Cape Coast Ghana; ^3^ Department of Agricultural Engineering University of Cape Coast Cape Coast Ghana

**Keywords:** β‐carotene, legumes, maize, porridge, protein

## Abstract

Addressing issues of protein and vitamin A deficiency remains vital for mitigating hunger and moderating malnutrition in many developing countries. In this study, maize porridge, which is widely consumed in several communities of low‐ to middle‐income countries, was experimented with as a vehicle for improving nutrient intake by adding soybean and ripe plantain to form composite flours. The physicochemical, functional, and proximate composition of the composite flours were determined, and the sensory properties and consumer acceptability of porridge prepared from the flours analyzed. Additionally, the willingness of respondents to consume the porridge was evaluated via questionnaire administration. The results show that adding soybean and ripe plantain led to increases in protein, iron, and β‐carotene, which affected water binding capacity, bulk density, solubility, and swelling power. The browning index reduced, leading to observable color changes while an increase in total soluble solids was observed. The sensory panelists were able to distinguish between the different types of porridge, but this did not affect the overall acceptability. Respondents revealed health and nutritional benefits as the major drivers for acceptability, and were willing to pay higher tokens for the newly developed composite porridge. This study provides a practical approach for creating nutritious and acceptable composite flours from locally available raw ingredients: maize, soybean, and ripe plantain, which can address moderate malnutrition relating to protein and vitamin A deficiency while enhancing food security.

## Graphical abstract

This study aims to enhance the nutritional quality of maize porridge, a popular meal in many developing countries, by adding soybean and ripe plantain flours which are rich in protein and β‐carotene, respectively. The addition of soybean and ripe plantain to form composite flour did not affect water binding, swelling power, and viscosity compared to maize flour. Sensory evaluation revealed high acceptability of the composite porridge, with panelists willing to pay higher tokens due to the health and nutritional benefits.

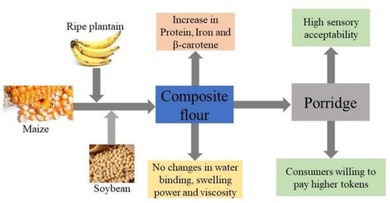

## 1 Introduction

Maize porridge is a staple across many African countries that is valued for its role as both a common breakfast meal and a crucial weaning food (Ekpa et al. 2019). The soft and easily digestible porridge is prepared by boiling maize flour in water, with sugar added to enhance palatability. The widespread consumption of maize porridge underscores its importance, providing a vital source of energy, especially for children at critical stages of growth and development (Ekpa et al. 2019). However, despite its ability to provide caloric needs, communities reliant on maize can become exposed to micronutrient deficiencies (hidden hunger), due to its inherent nutritional limitations. Maize contains low levels of β‐carotene (vitamin A precursor) and protein (Rouf Shah et al. 2016), therefore, consuming mainly maize‐based products can lead to vitamin A deficiency and protein‐energy undernutrition. To address this public health concern, supplementing maize flour with locally available and nutrient‐dense staples such as ripe plantain (rich in provitamin A carotenoids) and soybean (high in protein) can enhance nutritional quality and help to transform maize porridge into a more complete food.

The contribution of plantain (*Musa paradisiaca* L.) to food security is severely undermined by rapid post‐harvest deterioration, even though it is abundant in several tropical regions. The loss of plantain is particularly acute during bumper seasons, due to a combination of high perishability and limited processing technologies. The economic impact of this loss is huge, as Ayeh et al. (2023) reported weekly post‐harvest losses of about 174,000 USD in three satellite markets in Accra. This represents a profound loss of vital nutrients in addition to the economic consequences.

At the peak of ripening, plantain contains high levels of β‐carotene and iron, which according to Agbemafle et al. (2017) and Blomme et al. (2020), can contribute significantly to addressing dietary deficiencies, especially among vulnerable groups. Therefore, there is the need to create viable pathways for integrating ripe plantain into common foods such as maize porridge to reduce losses, enhance nutritional outcomes, and unlock economic opportunities.

To address the low protein content of maize porridge, available plant‐based proteins such as soybean need to be investigated. This is especially important due to the increasing interest in the integration and utilization of locally grown and readily accessible nutrient‐rich foods (Fetriyuna et al. 2023) to help end hunger and malnutrition (Sustainable Development Goal 2), while eradicating poverty (Sustainable Development Goal 1).

However, the consumer acceptability of new foods, albeit with improved nutritional qualities, could be faced with resistance because of changes in the cooking requirements, sensorial properties and probably higher cost compared to traditional foods. This could contribute to unwillingness among consumers to adopt new food habits (Costell et al. 2009). In this regard, determining the changes in nutritional content, sensory properties and consumer acceptability and the willingness to consume is paramount to the successful integration in the local diets of consumers.

In this study, composite maize flours were prepared by combining maize, soybean, and ripe plantain, and the nutritional and physicochemical properties were determined. Next, sensory evaluation of porridge prepared from the composite flours was carried out to evaluate the properties and consumer acceptability. Additionally, consumers were asked about the attributes of the maize porridge that influence the decision to consume, their knowledge about the nutritional benefits of soybean and ripe plantain, and the willingness to consume maize porridge containing soybean and ripe plantain.

## 2 Materials and Methods

### 2.1 Materials

The following chemicals were obtained from VWR Chemicals: acetone, ammonium thiocyanate, boric acid, ethylenediaminetetraacetic acid, Eriochrome black, ethanol, Folin‐Ciocalteu reagent, gallic acid, hydrochloric acid, hydrogen peroxide, hydroxylamine hydrochloride, iron (III) chloride, methanol, nitric acid, petroleum ether, potassium cyanide, potassium ferrocyanide, potassium permanganate, sodium bicarbonate, sodium hydroxide, sulfuric acid, and triethanolamine.

Maize (*Zea may*, Obaatanpa), soybean ( *Glycine max* , Jenguma), and ripe plantain ( *Musa x paradisiaca* , Apantu) were obtained from the Abura market in Cape Coast, Ghana, and authenticated by the Herbarium of the School of Biological Sciences, University of Cape Coast. Ripe plantain was in stage 5 of ripeness (Keri and Adel 2002).

The following instruments were used for the analysis: Atomic absorption spectrophotometer (Shimadzu, model 6401F), Brabender viscoamylograph (Type 801,203 W.G), color reader (CHN Spec, China, model CS‐10), hammer mill (Gratis Foundation, USA), pH meter (B10P Benchtop), refractometer (Milwaukee, model MA871), and spectrophotometer (Bibby Scientific Ltd., UK, Jenway 6400).

### 2.2 Preparation of Composite Flours and Quality Analysis

Maize, soybean, and ripe plantain were sorted and prepared into flours as follows: Maize (120°C for 40 min) and soybean (150°C for 45 min) were dried in an oven, cooled, and milled into flour according to a previously described procedure (Otoo et al. 2024). After peeling and slicing, ripe plantain was dried (60°C for 4 days) and milled into flour.

Seven composite flours were prepared by combining maize, soybean, and ripe plantain flours based on a simple lattice design with a minimum and maximum of 25% and 75%, respectively, for each component. Subsequently, quality characteristics of the composite flours were determined by analyzing the proximate composition, iron, and β‐carotene content. Furthermore, the pH, Brix, and color (color change and browning index) of the flours were analyzed.

The proximate composition of the flours was determined by analyzing the crude protein, ash, fat, and fiber. Using 2 g of flour, the protein content was determined based on Kjeldahl method using N x 6.25, while fat content was extracted with petroleum ether using the Soxhlet method. The defatted residue was subsequently acid and alkaline‐digested for the fiber content, while ash content was analyzed after burning in a furnace. The total carbohydrate and energy (calories) were estimated based on the difference and the Atwater factors, respectively. The obtained ash was used for the analysis of iron and calcium based on the atomic absorption spectrophotometer, while the levels of magnesium and potassium were obtained after titration (Nkansah et al. 2025).

The β‐carotene content of the flours was analyzed spectrophotometrically after extracting with acetone, while the Folin‐Ciocalteau method was used for the determination of total phenols (Otoo et al. 2024). The analysis of phytate and oxalate was carried out by titration against iron (III) chloride and potassium permanganate, respectively, while tannins were extracted with acetone and measured spectrophotometrically using Folin's reagent (Dadzie et al. 2025).

Using a Brabender viscoamylograph (Type 801,203 W.G), 40 g flour in 500 mL water was heated from 30°C to 95°C at a rate of 1.5°C/min, and the viscosity monitored at a velocity of 75 rpm (Otoo et al. 2024). The water and oil absorption were determined by dispensing flour (1 g) into water or oil, vortexed, centrifuged and decanted, and the differences in weight recorded. The bulk density of the flours was analyzed by measuring the tapped volume of 100 g flour placed in a 250 mL measuring cylinder. For the determination of swelling power, 1.0 g flour placed in 40 mL distilled water was heated in a water bath at 85°C with shaking for 30 min, allowed to cool to room temperature, centrifuged, decanted, and the difference in weight observed. The obtained supernatant was dried in an oven at 105°C for the determination of solubility (Kizzie‐Hayford et al. 2023).

### 2.3 Sensory Evaluation of Porridge

Porridge was prepared after selecting three of the composite flours consisting of 25% maize flour, 50% soybean, 25% plantain (M_25_S_50_P_25_), 25% maize, 25% soybean, 50% plantain (M_25_S_25_P_50_), and 33% each of maize flour, soybean, and plantain (M_33_S_33_P_33_). Then, the porridge was prepared by adding table salt (0.5 g) and sugar (3.5 g) to the flour (70 g), mixing with water (800 mL), and boiling for 15 min. A reference porridge sample was prepared from only maize and included for the sensory evaluation.

For assessing the consumer acceptability, 78 panelists were randomly recruited to rate the sensory qualities of the porridge by using a 9‐point hedonic scale ranging from dislike extremely (1) to like extremely (9) as described previously (Kizzie‐Hayford et al., 2023a). In another set of experiments, 15 new panelists were recruited for flash profiling to describe the sensory attributes and characteristics of the porridge using the procedures reported previously (Kizzie‐Hayford et al. 2024; Kizzie‐Hayford et al., 2023b).

### 2.4 Determination of Willingness to Consume Composite Porridge

After the sensory evaluation, panelists (*n* = 93) who participated in flash profiling and hedonic assessment of the porridge were asked to complete a four‐section questionnaire regarding the consumption of the traditional maize porridge and the newly formulated composite porridge. Section A of the questionnaire obtained information about the socio‐demographic characteristics, while Section B sought information regarding attributes that influence the consumption of maize porridge. In Sections C and D, the questions required respondents to indicate the level of knowledge on the nutritional benefits of soybean and ripe plantain, and the willingness to consume porridge prepared from the composite flours, respectively.

### 2.5 Data Analysis

Analysis of the quality attributes of the flours, and data obtained from the hedonic‐scale sensory evaluation was carried out in SPSS (IBM, SPSS Statistics 20) using ANOVA (analysis of variance) and the Tukey post hoc test. For comparative statistical purposes, maize flour and hence, maize porridge served as the control. Data obtained from the flash profiling was analyzed using the Senstools. Net software (OP&P Product Research BV, Utrecht, Netherlands) by carrying out Generalized Procrustes and principal component analyses.

The sociodemographic data obtained after administering the questionnaire were summarized using descriptive statistics. Attributes of the composite maize porridge that influence consumption were ranked using Kendall's Coefficient of Concordance (Kendall's W) (Legendre 2005).

To evaluate the acceptability of the porridge, two categories of statements related to the health/nutritional benefits of soybean and ripe plantain, and the willingness to buy maize porridge containing soybean and ripe plantain, were solicited. Using a 3‐point Likert scale of −1 (disagree), 0 (neither agree nor disagree), and 1 (agree) to proposed statements, the mean score for each statement was estimated as described previously (Ampofo‐Asiama et al., 2025). Significant differences among consumers who were willing or unwilling to consume the composite porridges based on their socio‐demographic characteristics were estimated using the (chi‐square) *χ* ^2^ test.

## 3 Results

### 3.1 Effects of Adding Soybean and Ripe Plantain on Nutrient and Mineral Content

Soybean flour (S_100_) had the highest protein (31.62 g/100 g) whilst ripe plantain (P_100_) and maize flour (M_100_) had the lowest (Table 1). Compared to the control (M_100_), significantly higher protein was observed in the composite flours containing M_25_:S_50_:P_25_ and M_29_:S_29_:P_41_, which had the highest and lowest protein content, respectively. A higher crude ash content was observed for both soybean and ripe plantain flours, contributing to significant increases in the composite flours. Soybean flour showed the highest fat content (12.45 ± 0.89 g/100 g) compared to maize and ripe plantain, depicting soybean as a major fat source in the composite flours. Similarly, the high fiber content of the composite flour, M_25_S_50_P_25_, can be explained by the high fiber content of soybean flour. The carbohydrate content of the composite flours was lower, although comparable energy values were observed with respect to maize flour.

**TABLE 1:** Proximate content (g/100 g) of maize (M), soybeans (S), ripe plantain (P) and composite flours. Subscripts represent the fraction of each component in the flour.

Formulation	Protein	Ash	Fat	Fiber	Carbohydrates	Energy (kcal/g)
M_100_	7.92 ± 0.07^a^	1.35 ± 0.03^a^	5.01 ± 0.06^a^	4.52 ± 0.27^a^	81.20 ± 0.29^a^	392.53 ± 1.41^a^
S_100_	31.62 ± 1.04^b^	3.82 ± 0.08^b^	12.45 ± 0.89^b^	8.65 ± 1.21^b^	43.46 ± 1.83^b^	395.07 ± 11.8^ab^
P_100_	6.04 ± 0.15^c^	3.61 ± 0.14^b^	1.32 ± 0.07^c^	1.81 ± 0.05^c^	87.22 ± 0.22^c^	381.3 ± 1.25^b^
M_29_: S_41_: P_29_	20.31 ± 0.34^d^	3.17 ± 0.02^c^	8.7 ± 0.08^d^	4.22 ± 0.20^a^	63.60 ± 0.40^d^	405.5 ± 2.26^b^
M_29_: S_29_: P_41_	15.69 ± 0.27^e^	2.58 ± 0.39d	6.78 ± 0.20^e^	5.62 ± 0.16^d^	69.33 ± 0.54^e^	389.86 ± 3.03^a^
M_25_: S_25_: P_50_	16.48 ± 0.20^e^	2.61 ± 0.22^d^	6.11 ± 0.00^e^	3.54 ± 0.16^a^	71.26 ± 0.34^e^	398.87 ± 1.60^a^
M_41_: S_29_: P_29_	17.59 ± 0.34^e^	2.64 ± 0.09^d^	6.75 ± 0.02^e^	5.54 ± 0.27^d^	67.48 ± 0.44^e^	389.95 ± 2.31^a^
M_33_: S_33_: P_33_	17.81 ± 0.16^e^	2.79 ± 0.07^d^	7.39 ± 0.14^f^	4.76 ± 0.03^a^	67.25 ± 0.23^e^	397.23 ± 1.68^a^
M_50_: S_25_: P_25_	17.15 ± 0.17^e^	2.77 ± 0.07^d^	5.22 ± 0.05^a^	5.38 ± 0.27^a^	69.48 ± 0.33^e^	382.74 ± 1.64^b^
M_25_: S_50_: P_25_	23.53 ± 0.16^f^	3.53 ± 0.03^b^	9.78 ± 0.12^g^	6.29 ± 0.36^e^	56.87 ± 0.41^f^	397.04 ± 2.20^a^

*Note:* Values in the same column with different superscripts are significantly different (*p* < 0.05).

The iron content of the composite flours was significantly higher than maize flour due to the high levels observed in ripe plantain (Table 2). Among the composite flours, M_25_:S_25_:P_50_, which had the highest ripe plantain portion, had the highest iron content of 1.19 g/100 g. No significant differences in calcium and magnesium were observed between the composite flours and maize flour; however, with the exception of M_41_:S_29_:P_29_, the potassium content of the composite flours was higher than maize flour due to the high level observed in ripe plantain (Table 2).

**TABLE 2:** Minerals content maize (M), soybean (S), ripe plantain (P) and composite flours.

Sample	Iron	Calcium	Magnesium	Potassium
	(mg/100g)	(mg/100 g)	(mg/100 g)	(mg/100 g)
M_100_	0.39 ± 0.01^a^	0.92 ± 0.02^a^	0.11 ± 0.01^a^	0.36 ± 0.02^a^
S_100_	0.31 ± 0.01^b^	0.93 ± 0.01^a^	0.08 ± 0.02^b^	0.48 ± 0.04^b^
P_100_	1.98 ± 0.03^c^	1.22 ± 0.06^b^	0.10 ± 0.01^a^	1.21 ± 0.02^c^
M_29_:S_41_:P_29_	0.59 ± 0.01^d^	1.01 ± 0.10^a^	0.11 ± 0.01^a^	0.55 ± 0.01^b^
M_29_:S_29_:P_41_	1.00 ± 0.03^e^	0.96 ± 0.09^a^	0.10 ± 0.01^a^	0.84 ± 0.02^d^
M_25_:S_25_:P_50_	1.19 ± 0.03^f^	0.97 ± 0.10^a^	0.10 ± 0.01^a^	0.89 ± 0.02^d^
M_41_:S_29_:P_29_	0.75 ± 0.03^g^	0.95 ± 0.07^a^	0.10 ± 0.02^a^	0.44 ± 0.02^a^
M_33_:S_33_:P_33_	0.79 ± 0.02^g^	0.99 ± 0.08^a^	0.11 ± 0.01^a^	0.62 ± 0.01^e^
M_50_:S_25_:P_25_	0.68 ± 0.02^g^	0.96 ± 0.08^a^	0.11 ± 0.01^a^	0.53 ± 0.02^b^
M_25_:S_50_:P_25_	0.62 ± 0.03^d^	0.93 ± 0.08^a^	0.11 ± 0.01^a^	0.61 ± 0.01^e^

*Note:* Values in the same column with different superscripts are significantly different (*p* < 0.05).

### 3.2 Influence of Adding Soybean and Ripe Plantain on Phytochemical and Physicochemical Quality

The high levels of β‐carotene in ripe plantain (Table 3) resulted in an increase in the composite flours compared to maize flour, with the highest content of 2.32 ± 0.13 mg/100 g observed in M_25_:S_25_:P_50_. However, the composite flours with low portions of ripe plantain did not show any significantly higher β‐carotene levels compared to maize flour. The composite flours had lower phenolic content; on the contrary, higher phytate, oxalate, and tannins were observed compared to maize flour.

**TABLE 3:** Phytochemical content (mg/100 g) of maize (M), soybean (S), ripe plantain (P), and composite flours.

Formulation	β‐Carotene	Phenolic content	Phytate	Oxalate	Tannins
M_100_	1.13 ± 0.12^a^	0.32 ± 0.02^a^	2.30 ± 0.40a	0.20 ± 0.00^a^	0.27 ± 0.10^a^
S_100_	0.60 ± 0.14^b^	0.22 ± 0.02^b^	10.20 ± 0.90^b^	0.40 ± 0.10^b^	0.45 ± 0.10^b^
P_100_	4.21 ± 0.31^a^	0.30 ± 0.02^a^	0.90 ± 0.10^c^	0.20 ± 0.00^a^	0.27 ± 0.10^a^
M_29_: S_41_: P_29_	1.90 ± 0.10^d^	0.28 ± 0.01^a^	3.47 ± 0.40^a^	0.36 ± 0.00^c^	0.35 ± 0.01^a^
M_29_: S_29_: P_41_	2.18 ± 0.12^e^	0.22 ± 0.01^b^	3.15 ± 0.60^a^	0.58 ± 0.02^d^	0.38 ± 0.01^a^
M_25_: S_25_: P_50_	2.32 ± 0.13^e^	0.27 ± 0.02^a^	5.18 ± 1.05^d^	0.24 ± 0.01^e^	0.37 ± 0.01^a^
M_41_: S_29_: P_29_	1.69 ± 0.13^a^	0.23 ± 0.01^b^	3.65 ± 0.72^a^	0.41 ± 0.01^b^	0.35 ± 0.01^a^
M_33_: S_33_: P_33_	1.94 ± 0.13^d^	0.24 ± 0.01^b^	3.60 ± 0.82^a^	0.25 ± 0.01^e^	0.34 ± 0.00^a^
M_50_: S_25_: P_25_	1.63 ± 0.10^a^	0.25 ± 0.01^a^	3.52 ± 0.80^a^	0.23 ± 0.01^e^	0.26 ± 0.01^a^
M_25_: S_50_: P_25_	1.72 ± 0.10^a^	0.23 ± 0.01^b^	5.25 ± 0.52^d^	0.13 ± 0.01^f^	0.39 ± 0.01^a^

*Note:* Values in the same column with different superscripts are significantly different (*p* < 0.05).

The pH of the composite flours was not significantly different from maize flour with the exception of M_29_:S_41_:P_29_ and M_25_:S_50_:P_25_ which recorded significantly higher values (Table 4). The total soluble solids of the composite flours (range of 1.10°–1.40°Brix) were all higher than maize flour (0.40° ± 0.08°Brix) due to the addition of ripe plantain (3.02° ± 0.08°Brix). The browning index of maize flour was 48.70 ± 6.35, while the composite flours ranged from 19.54–34.85, showing the effect of adding soybean (18.54 ± 1.37) and ripe plantain (28.65 ± 52). Using maize flour as reference, a color change of 8.64 to 11.65 was recorded for the composite flours (Table 4).

**TABLE 4:** Physicochemical properties of maize (M), soybean (S), ripe plantain (P), and composite flours.

Sample	pH	Brix	Color change	Browning index
M_100_	5.39 ± 0.11^a^	0.40 ± 0.08^a^		48.70 ± 6.35^a^
S_100_	6.29 ± 0.08^b^	3.02 ± 0.08^b^		18.54 ± 1.37^b^
P_100_	4.62 ± 0.11^c^	1.50 ± 0.07^c^		28.65 ± 3.52^c^
M_29_: S_41_: P_29_	5.65 ± 0.01^d^	1.30 ± 0.00^d^	11.65 ± 1.11	21.89 ± 2.31^d^
M_29_: S_29_: P_41_	5.43 ± 0.01^a^	1.40 ± 0.01^d^	9.65 ± 0.98	19.54 ± 2.62^b^
M_25_: S_25_: P_50_	5.37 ± 0.01^a^	1.40 ± 0.00^d^	8.65 ± 0.91	28.52 ± 2.48^c^
M_41_: S_29_: P_29_	5.52 ± 0.00^a^	1.30 ± 0.00^d^	8.64 ± 0.87	24.66 ± 2.14^e^
M_33_: S_33_: P_33_	5.54 ± 0.01^a^	1.30 ± 0.00^d^	9.65 ± 0.72	29.59 ± 2.23^c^
M_50_: S_25_: P_25_	5.51 ± 0.02^a^	1.20 ± 0.01^d^	11.65 ± 1.05	34.85 ± 1.63^f^
M_25_: S_50_: P_25_	5.79 ± 0.01^e^	1.10 ± 0.00^d^	10.37 ± 1.00	31.58 ± 2.91^g^

*Note:* Values in the same column with different superscripts are significantly different (*p* < 0.05).

### 3.3 Changes in Functional and Viscographic Properties

Table 5 shows that the water absorption capacity of the composite flours (range of 2.26–2.65 g/g) was significantly lower than maize flour (2.97 ± 0 0.05 g/g), while with the exception of M_29_:S_41_:P_29_ (2.30 ± 0.15), no significant difference was observed between the oil absorption capacity of the composite flours (range of 2.04–2.11 g/g) and maize flour (1.96 ± 0.09). The bulk density of maize, soybean, and ripe plantain flours also showed no significant differences, and consequently, there were no differences in that of the composite flours compared to maize flour. Maize flour (5.39 ± 0.25 g/g) had a high solubility compared to ripe plantain (5.01 ± 0.21 g/g) and soybean (3.27 ± 0.15 g/g) flours, leading to lower solubility of the composite flours. The composite flours had high swelling power compared to maize flour due to the higher values of soybean and ripe plantain.

**TABLE 5:** Functional properties of maize (M), soybean (S), ripe plantain (P), and the composite flours.

Sample	Water absorbed	Oil absorbed	Bulk density	Solubility index		Swelling power
	(g/g)	(g/g)	(mg/L)		(%)	(g/g)
M_100_	2.97 ± 0.05^a^	1.96 ± 0.09^a^	0.75 ± 0.09^a^	5.39 ± 0.75^a^		4.34 ± 0.26^a^
S_100_	2.47 ± 0.02^b^	2.34 ± 0.02^b^	0.65 ± 0.07^a^	3.27 ± 0.55^b^		5.27 ± 0.16^b^
P_100_	1.32 ± 0.08^c^	0.58 ± 0.07^c^	0.79 ± 0.10^a^	5.01 ± 0.85^a^		5.82 ± 0.21^c^
M_29_:S_41_:P_29_	2.42 ± 0.10^b^	2.30 ± 0.15^b^	0.76 ± 0.01^a^	4.32 ± 0.22^a^		4.78 ± 0.32^a^
M_29_:S_29_:P_41_	2.36 ± 0.13^b^	2.11 ± 0.14^a^	0.68 ± 0.01^b^	4.01 ± 0.16^b^		5.15 ± 0.30^b^
M_25_:S_25_:P_50_	2.35 ± 0.11^b^	2.05 ± 0.12^a^	0.73 ± 0.01^a^	4.54 ± 0.21^a^		5.49 ± 0.22^d^
M_41_:S_29_:P_29_	2.45 ± 0.13^b^	2.07 ± 0.15^a^	0.71 ± 0.01^a^	4.85 ± 0.13^a^		5.55 ± 0.40^d^
M_33_:S_33_:P_33_	2.43 ± 0.16^b^	2.04 ± 0.18^a^	0.76 ± 0.01^a^	4.43 ± 0.17^a^		5.12 ± 0.19^b^
M_50_:S_25_:P_25_	2.65 ± 0.09^d^	2.08 ± 0.12^a^	0.75 ± 0.02^a^	4.01 ± 0.21^b^		5.74 ± 0.24^c^
M_25_:S_50_:P_25_	2.26 ± 0.08^b^	2.08 ± 0.10^a^	0.71 ± 0.01^a^	4.00 ± 0.14^b^		5.20 ± 0.19^b^

*Note:* Values in the same column with different superscripts are significantly different (*p* < 0.05).

The pasting properties of the flours shown in Table 6 reveal the final viscosity of the composite flours ranged from 1747 to 1917 BU compared to 1396 ± 167.86 BU observed for maize flour. A peak time of 9.92–10.41 min was observed for the composite flours compared to 10.53 ± 1.01 observed for maize flour. Overall, with the exception of breakdown viscosity, no significant difference was observed between the composite flours and maize flour with respect to the measured pasting properties.

**TABLE 6:** Pasting properties of maize (M), soybean (S), ripe plantain (P), and the composite flours.

Sample	Pasting temperature	Peak viscosity	Breakdown viscosity	Trough viscosity	Setback viscosity	Final viscosity	Peak time
M_100_	81.25 ± 7.25^a^	1082 ± 66.76^a^	40 ± 8.69^a^	1042 ± 63.55^a^	354 ± 14.2^a^	1396 ± 167.86^a^	10.53 ± 1.01^a^
S_100_	85.54 ± 6.40^a^	1305 ± 96.44^b^	124 ± 18.46^b^	1181 ± 45.44^a^	325 ± 25.37^a^	1561 ± 87.97^a^	10.43 ± 0.93^a^
P_100_	78.62 ± 7.82^a^	1125 ± 105.9^a^	218 ± 13.2^c^	907 ± 81.18^a^	456 ± 15.9^b^	2452 ± 107.85^a^	9.25 ± 1.10^a^
M_29_: S_41_: P_29_	80.99 ± 9.25^a^	1169 ± 73.75^a^	127 ± 23.2^b^	1042 ± 97.65^a^	370 ± 16.16^a^	1750 ± 214.87^a^	10.41 ± 0.90^a^
M_29_: S_29_: P_41_	82.05 ± 10.85^a^	1215 ± 99.72^a^	153 ± 14.78^b^	1062 ± 47.99^a^	360 ± 55.21^a^	1747 ± 104.90^a^	10.09 ± 0.90^a^
M_25_: S_25_: P_50_	81.06 ± 8.41^a^	1098 ± 109.5^a^	89 ± 12.68^d^	1009 ± 96.88^a^	389 ± 18.85^a^	1805 ± 87.64^a^	9.92 ± 1.02^a^
M_41_: S_29_: P_29_	82.05 ± 7.67^a^	1108 ± 75.86^a^	86 ± 6.254^d^	1023 ± 88.69^a^	372 ± 24.15^a^	1754 ± 97.37^a^	10.36 ± 1.42^a^
M_33_: S_33_: P_33_	81.19 ± 10.92^a^	1157 ± 86.16^a^	112 ± 10.28^b^	1045 ± 106.6^a^	379 ± 24.97^a^	1718 ± 107.79^a^	10.08 ± 1.89^a^
M_50_: S_25_: P_25_	81.92 ± 8.49^a^	1152 ± 87.69^a^	123 ± 8.686^b^	1029 ± 74.99^a^	368 ± 22.33^a^	1733 ± 85.03^a^	9.99 ± 1.48^a^
M_25_: S_50_: P_25_	81.58 ± 8.81^a^	1107 ± 81.97^a^	97 ± 7.237^d^	1010 ± 86.13^a^	397 ± 49.85^a^	1917 ± 126.67^a^	9.98 ± 0.78^a^

*Note:* Values in the same column with different superscripts are significantly different (*p* < 0.05).

### 3.4 Sensory Acceptability of Composite Porridges

**FIGURE 1:**
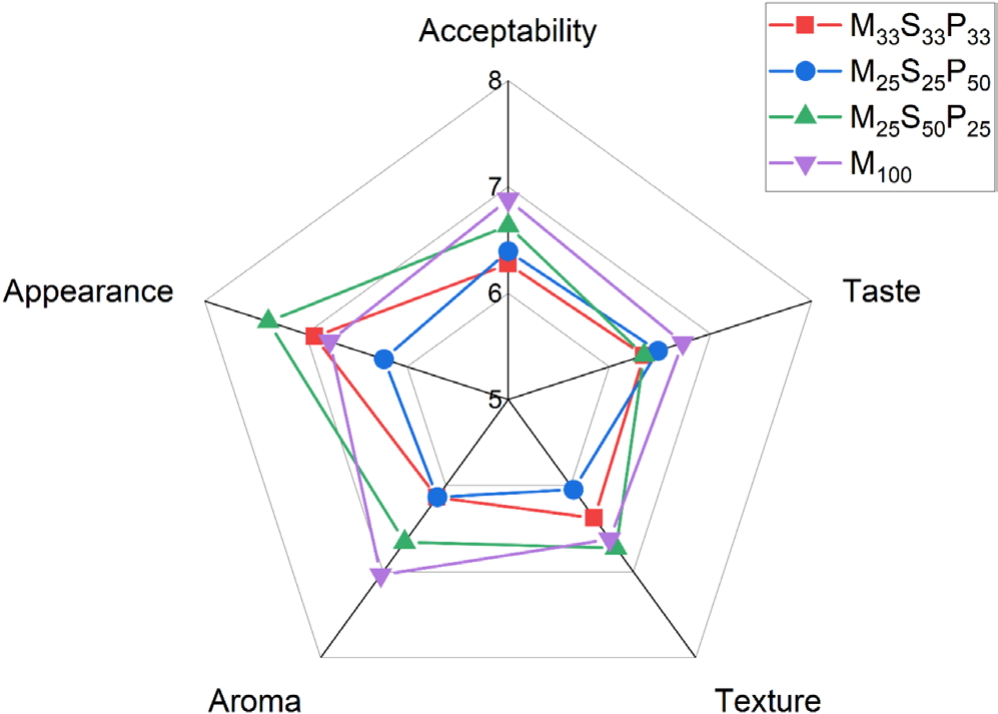
Hedonic sensory scores of porridges prepared from roasted maize (M_100_) and three selected composite flours M, S and P represent roasted maize, soybeans, ripe plantain flours, respectively. Subscripts represent the fraction of each component in the composite flour.

The sensory evaluation scores for appearance ranged between 6.23–7.37, with porridge prepared from the composite flours M_25_S_25_P_50_ and M_25_S_50_P_25_‐ having the highest and lowest scores, respectively (Figure 1). The scores for taste ranged between 6.34–6.73, with porridge prepared from maize flour having the highest score. With respect to texture, a range of 6.05–6.73 was observed, with M_33_S_33_P_33_ and M_25_S_50_P_25_ having the lowest and highest scores, respectively. The score for aroma was in the range of 6.13–7.03, with maize flour showing the highest value. The overall acceptability scores, which ranged from 6.27–6.88, were highest and lowest for porridges prepared from maize flour and M_33_S_33_P_33_, respectively.

**FIGURE 2:**
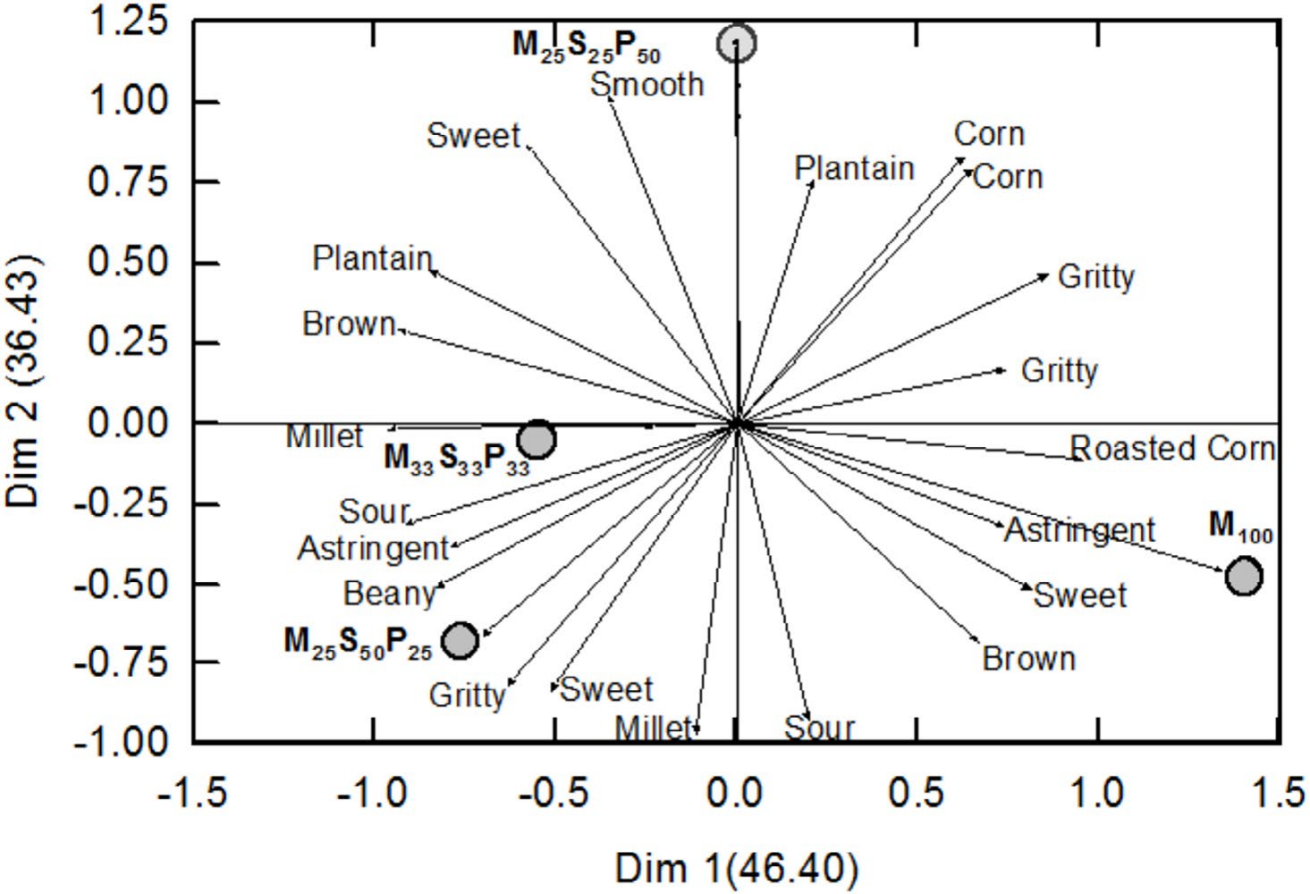
Generalized Procrustes Analyses plot of descriptors for porridge prepared from roasted maize flour (M_100_) and three selected composite flours M, S and P represent roasted maize, soybeans, ripe plantain flours, respectively. Subscripts represent the fraction of each component in the composite flour.

The GPA plot (Figure 2) of the flash profiling sensory evaluation shows the panelists could distinguish between porridge prepared from maize flour and the composite flours. The group descriptors reveal that porridge from maize flour was frequently described as *brown* and *astringent* with a *roasted corn* taste, while porridge from M_25_S_25_P_50_ was described as *smooth* with a *plantain* aroma. On the other hand, M_33_S_33_P_33_ was described as *sour* with a *millet* taste, while M_25_S_50_P_25_ was considered *beany* and *gritty*.

### 3.5 Attributes That Influence Maize Porridge Consumption and Willingness to Consume the Composite Porridge

Table 7 shows the demographic distribution of the panelists, 60% of whom were males with a similar number being of the Akan ethnic group. About 90% were Christians, 10% were Moslems, close to 70% were single, while 20% were married. With respect to status within the family, about 20% were either a spouse or bread winner of family, while 77% were considered children. The panelists were either students (69%) or public workers (31%) and all of whom (100%) had some knowledge about or consumed maize porridge.

**TABLE 7:** Socio‐demographic characteristics of respondents.

Characteristic	Frequency	Percentage (%)
Gender		
Male	57	61.29
Female	36	38.71
Ethnicity		
Akan	56	60.22
Ga	14	15.05
Ewe	12	12.90
Guan	4	4.30
Other	7	7.53
Religion		
Christian	84	90.32
Muslim	9	9.68
Traditional	0	0.00
Others	0	0.00
Marital status		
Single	72	77.42
Married	20	21.51
Divorced	1	1.08
Status in household		
Bread winner	11	11.83
Spouse	10	10.75
Child	72	77.42
Occupation		
Worker	29	31.18
Student	64	68.82
Aware of roasted maize porridge		
Aware	93	100.00
Unaware	0	0.00
Consumes roasted maize porridge		
Yes	93	100.00
No	0	0.00
Enjoys roasted maize porridge		
Yes	85	91.40
No	8	8.60

The respondents were aged 27.15 (±9.98) years with a minimum and maximum of 18.00 and 51.00 years, respectively. More than 60% of the consumers were students who were less than 25 years of age. A minimum and maximum monthly income of 300 and 13,000 (ȼ) was reported, respectively, while the number of years of formal education ranged between 14 to 24 years, showing that the panelists had at least some tertiary education.

On the attributes that influenced the consumption of maize porridge, health, with a mean score of 1.60, was the major reason driving porridge consumption (Table 8). Other important attributes included nutrient (mean score 1.84) and taste (mean score 3.52), with weight control (mean score 8.85) and price (mean score 8.97) as the lowest in importance.

**TABLE 8:** Ranking of attributes that influence the consumption of maize porridge.

Attribute	Mean rank	Rank
Health	1.60	1st
Nutrient	1.84	2nd
Taste	3.52	3rd
Sweetness	3.73	4th
Appearance	5.66	5th
Texture	6.45	6th
Color	6.15	7th
Variety	8.42	8th
Weight	8.85	9th
Price	8.97	10th
Number of observations	93	
Kendall's W	0.83	
Chi‐squared	697.80	
Df	9	
Assymp. Sig.	0.00	

The perception about the nutritional/health benefits of soybean and ripe plantain, and the potential for purchasing the composite flours (Table 9) depicts that about 80% of panelists agreed that soybean and ripe plantain can help increase the intake of protein and β‐carotene and iron, respectively. With respect to purchasing potential, about 47% of panelists agreed that the composite flours will likely be more expensive. On the other hand, about 65% agreed to choose the composite flours over other flours on the market if they sell at the same price. About 61% of the panelists were willing to consume the composite flours if they are well packaged, well labeled and the nutritional content displayed on the product.

**TABLE 9:** Health/nutritional and purchasing perception index of maize porridge.

Statement	Agree (+1)	Neutral (0)	Disagree (−1)	Mean score
*Health/Nutritional Statement*				
(a) Soybean can help increase protein intake	72	12	9	0.68
(b) Ripe plantain can help boost iron and vitamin A intake	65	11	17	0.52
(c) Ripe plantain and soybeans can help reduce malnutrition	66	16	11	0.59
(d) Ripe plantain has a lower glycemic index and is a healthy choice for diabetics	52	28	13	0.42
(e) Ripe plantain contains resistant starch which can help reduce weight	57	25	11	0.49
Average health/nutritional perception index				0.54
*Purchasing statement*				
(f) Roasted maize flour containing ripe‐plantain and soybean is likely to be more expensive	44	16	33	0.12
(g) I will choose roasted maize flour containing ripe plantain and soybeans over other flours on the market if they all sell at the same price	61	7	25	0.39
(h) I am willing to pay a little high for roasted maize flour containing ripe plantain and soybean due to the health and nutritional benefits	57	9	27	0.32
(i) I will be willing to buy roasted maize flour containing ripe plantain and soybean if it is well packaged	62	10	21	0.44
(j) I will be willing to buy roasted maize flour containing ripe plantain and soybean if it is well labeled	57	14	22	0.38
(k) I will be willing to buy roasted maize flour containing ripe plantain and soybean if the nutritional content is displayed on the product	61	13	19	0.45
Purchasing perception index				0.35
*Overall average perception index*				0.44

Table 10 shows the panelists' willingness to consume composite porridge based on the socio‐demographic characteristics. Out of the panelists, 83 representing about 90% were willing to consume the porridge. With respect to sex, although a non‐significant effect was observed, a higher percent of males were willing to consume the porridge. Similarly, a non‐significant effect of marital status, status of the consumer within the family, and occupation was observed.

**TABLE 10:** Characteristics of consumers who were willing (WTC) or unwilling (UWTC) to consume composite porridge.

	WTP	UWTP	*χ* ^2^	*p*
Sex			2.14	0.14
Male	53 (93)	4 (7)		
Female	30 (83.3)	6 (16.7)		
Marital status			8.99	0.10
Single	64 (88.90)	8 (11.11)		
Married	19 (95.00)	1 (5.00)		
Divorced	0	1 (100)		
Status in family			1.47	0.48
Head	10 (90.90)	1 (9.10)		
Spouse	10 (100)	0		
Child	63 (87.50)	9 (12.50)		
Occupation			2.34	0.13
Worker	28 (96.60)	1 (3.40)		
Student	55 (85.90)	9 (14.10)		
Enjoys roasted maize porridge			0.03	0.867
Yes	76 (89.40)	9 (10.60)		
No	7 (87.50)	1 (12.50)		

## 4 Discussion

Incorporating soybean increased protein content to an average of 18.40%, showing that consuming 100 g of the composite flours can help address protein malnutrition in children less than 3 years who have a recommended daily allowance (RDA) of 13 g/day (Trumbo et al. 2002). However, the composite flours‐ M_29_:S_41_:P_29_ and M_25_:S_50_:P_25_‐ with protein levels greater than 20 g/100 g can help meet the protein needs of children aged 4–8 years who require about 19 g protein per day. Considering the widespread prevalence of protein malnutrition in most rural African communities, the addition of soybean to maize porridge offers the opportunity to tackle this deficiency.

The high ash content of the composite flours, due to the contribution from soybean and ripe plantain, shows an increase in mineral composition. The high fat content of soybean leads to an increase in the composite flours that can contribute to the caloric energy which can be beneficial to consumers such as children who need lipid energy for growth and development (Trumbo et al. 2002). Conversely, the increase in fat can enhance lipid oxidation which might affect shelf life, necessitating possible changes in packaging and storage. The comparable fiber contents show that consumers will not lose the health promoting benefits of fiber when substituting maize with the composite flours.

The high iron and potassium content of the composite flours can help enhance intake, while comparable calcium and magnesium levels will be achieved when consuming the composite flours. The increased β‐carotene content of the composite flours due to the contribution from ripe plantain can help improve β‐carotene intake. Taking a conversion rate of 12 mg β‐carotene to 1 mg retinol (Tang 2010), the composite flours can provide an average of 159.2 mg of Retinol (RAE), which can help meet about 40% of the RDA in children less than 8 years.

The comparable pH levels indicate minimal differences in sourness between the composite and maize flours. However, the lower browning index of the composite flours may explain the high values observed for color change. This color change may be noticeable to consumers as it surpasses the perceptible threshold of 6 based on the scale developed by Cserhalmi et al. (Nkansah et al. 2025).

The similarities in the functional and viscographic properties of the composite to maize flour show that similar conditions can be employed during processing, packaging and storage. This is important as most consumers may be familiar with the cooking behavior of maize flour and may not have to adapt to new techniques when preparing the composite porridges. Further, the properties of the composite flours and prepared porridges may not differ from maize with respect to weight, volume, texture and viscosity.

The addition of soybean and ripe plantain affected the sensory attributes of the porridge, as could be seen in the different descriptors generated. Clearly, the sensory panel could identify differences in porridge appearance as was corroborated by the estimates of the color change. Adding ripe plantain flour, which has a bright yellow color, enhanced the appearance of the composite porridges. However, the intensity of roasted corn taste was lowered, while the taste of plantain and the beany flavor of soybean were perceived. The high consumer acceptance of maize porridge compared to the composites can be attributed to familiarity, which agrees with the observation of Baker et al. (2022), who showed that consumers are more inclined to consume foods with familiar taste. It is possible that the lower overall acceptability of the composite porridges could be due to the beany flavor impacted by the addition of soybean; therefore, strategies such as fermentation and sprouting (Kizzie‐Hayford et al. 2025) can be explored to reduce the beany flavor.

The high estimated Kendall's Coefficient of Concordance reveals a strong agreement among the panelists in ranking the attributes of maize porridge, showing consistency in preferences and perceptions, possibly due to the popularity of maize porridge. The choice of health and nutrients being the most important factors influencing maize porridge consumption aligns with an increasing global trend where consumers have become more health‐conscious, leaning towards functional and nutritious foods (Baker et al. 2022). The popularity of maize porridge may stem from its reputation as a traditional and wholesome; however, this popularity as well as the ease of accessibility could explain why price was viewed as the least important factor influencing consumption. This finding, nevertheless, contrasts with the report of Lusk and Briggeman (2009), who observed that price plays a significant role in consumer decision‐making. Similarly, the low ranking of weight suggests that packaging or portion size is not a major concern because maize porridge, which is often prepared at home, has little need for packaging.

An estimated average selling price of 12.28 ȼ/kg was obtained for the composite flours compared to 10.70 ȼ/kg for maize flour. This 15% increase in price may prove more affordable compared to the 50% observed when Bambara groundnut (Otoo et al. 2024) was used as a protein source. This shows that soybean presents a more cost‐effective protein alternative to Bambara groundnut, as consumers exhibit greater willingness to adopt nutritious alternatives when price barriers are minimized (Alsubhi et al. 2022).

The positive perception about the benefits of soybean reflects a growing awareness about its role as a good plant‐based protein source that can be used to enhance protein intake in regions where animal protein is expensive (Messina 2022). On the contrary, the limited awareness about the nutritional and functional properties of ripe plantain highlights a gap in consumer knowledge, considering that ripe plantain contains resistant starch, with a low glycemic index (Udo et al. 2021) that can help regulate blood sugar while promoting gut health (Oluwajuyitan and Ijarotimi 2019).

The willingness of the panelist to consume the composite porridges may be attributed to the familiarity of the ingredients and their perceived nutritional benefits. The observation that socio‐demographic factors do not influence willingness to consume is consistent with the findings of Verbeke (2005), who observed that factors such as taste, nutritional value, and familiarity often outweigh socio‐demographic characteristics in determining the acceptability of food. Interestingly, whether a consumer enjoys eating maize porridge or not did not influence willingness to consume, suggesting that the composite porridge may be perceived as being distinct from traditional maize porridge, possibly due to their unique composition and nutritional profile.

## 5 Conclusions

Addressing issues of protein energy undernutrition, as well as vitamin A and iron deficiencies can be achieved by using existing, well‐known foods, such as maize porridge, as vehicles for introducing nutrient‐rich staples. The addition of soybean and ripe plantain, which are respectively high in protein and β‐carotene, successfully increased the levels of the respective nutrients in the composite flour. Although consumers perceived changes in the sensory property of the composite porridge, there were no major differences in acceptability, with health and nutrient concerns being the main factors considered to drive willingness to consume. Future studies to improve the quality of the composite flour, such as reduction in the beany flavor of soybean, could be helpful for enhancing consumer acceptability.

## 6 Highlight

This study demonstrates that enriching popular maize porridge with local raw materials‐soybean and ripe plantain‐ produces a nutritious meal capable of addressing moderate malnutrition associated with deficiencies in protein, iron, and vitamin A.

## 7 Limitation

One major limitation is the exclusion of children from sensory analysis. This is a critical gap, as children are the ultimate end‐users who would derive the greatest health benefit from the nutritionally improved porridge.

## Author Contributions

**Salifu Seidu‐Larry:** data curation (equal), methodology (equal), validation (equal). **Claudia Asantewaa Gyimah:** investigation (equal), methodology (equal), resources (equal). **Vivianne Geraldo:** investigation (equal), methodology (equal), writing – original draft (equal). **Isaaca Adade:** methodology (equal), validation (equal), visualization (equal).

## Funding

The authors have nothing to report.

## Conflicts of Interest

The authors declare no conflicts of interest.

## Data Availability

The data that support the findings of this study are available on request from the corresponding author.
